# Supplementary material for: Comprehensive clinical assessment identifies specific neurocognitive deficits in working-age patients with long-COVID
Source: PLoS One. 2022 Jun 10;17(6):e0267392. doi: 10.1371/journal.pone.0267392 (PMC9187094; doi:10.1371/journal.pone.0267392)
Supplement: S1 Table — (DOCX) [file pone.0267392.s002.docx]

**Supplementary Table 1 – Common symptoms, patient reported outcome measures, cognitive testing scores and functional status in patients hospitalised by acute illness vs. managed in the community.**

|  | | Hospitalised | Community | Difference | Significance |
| --- | --- | --- | --- | --- | --- |
| Symptom free | | 24.5% | 13.2% | 11.3% | χ^2^ p = 0.052 |
| Symptoms (all symptoms occurring in ≥25%)† | |  |  |  |  |
| Any shortness of breath (*out of 3*) | | 0.85 (±1.01) | 1.11 (±1.0) | 0.26 | 0.087 |
|  | on moderate activity | 0.47 (±0.5) | 0.56 (±0.5) | 0.09 | 0.34 |
|  | on mild activity | 0.25 (±0.43) | 0.41 (±0.49) | 0.16 | **0.046** |
|  | at rest | 0.13 (±0.34) | 0.14 (±0.35) | 0.01 | >0.9 |
| Fatigue |  | 0.45 (±0.5) | 0.57 (±0.5) | 0.12 | 0.15 |
| Any cognitive symptoms (*out of 4*) | | 0.68 (±1.05) | 1.14 (±1.34) | 0.46 | **0.049** |
|  | poor concentration | 0.34 (±0.48) | 0.42 (±0.5) | 0.08 | 0.33 |
|  | poor memory | 0.21 (±0.41) | 0.34 (±0.48) | 0.13 | 0.084 |
|  | poor attention | 0.08 (±0.27) | 0.31 (±0.46) | 0.23 | **<0.001** |
|  | confusion | 0.06 (±0.23) | 0.07 (±0.25) | 0.01 | >0.9 |
| Muscle aches |  | 0.26 (±0.45) | 0.32 (±0.47) | 0.06 | 0.49 |
| Low mood |  | 0.23 (±0.42) | 0.30 (±0.46) | 0.08 | 0.38 |
| Difficulty getting to sleep | | 0.11 (±0.32) | 0.34 (±0.48) | 0.23 | **0.002** |
| Difficulty staying asleep | | 0.23 (±0.42) | 0.29 (±0.46) | 0.06 | 0.47 |
| Anxiety |  | 0.26 (±0.34) | 0.28 (±0.45) | 0.02 | 0.56 |
| Exercise intolerance |  | 0.17 (±0.38) | 0.28 (±0.45) | 0.11 | 0.14 |
| Patient reported outcome measures | |  |  |  |  |
| GAD-7 Anxiety (0-21) | | 4.36 (±4.05) | 5.69 (±5.32) | 1.33 | 0.196 |
| PHQ-9 Depression (0-27) | | 6.21 (±5.46) | 7.59 (±5.52) | 1.38 | 0.092 |
| PCL-5 Post-traumatic stress (0-80) | | 12.9 (±14.6) | 13.3 (±14.1) | 0.40 | 0.522 |
| EQ5D Quality of life (0-100) | | 65.9 (±75) | 60.6 (±67) | -5.30 | 0.059 |
| Fatigue assessment scale 10-50 | | 22.3 (±8.3) | 25.3 (±8.3) | 3.00 | **0.029** |
| NIH Cognitive testing battery | |  |  |  |  |
| Crystallised composite | | 54.8 (±10.7) | 57.8 (±10.1) | 3.00 | 0.071 |
| Fluid composite | | 53.3 (±12.3) | 52 (±12.9) | -1.30 | 0.552 |
| Total composite | | 54.5 (±11.7) | 55.8 (±10.8) | 1.30 | 0.505 |
| WHO Performance status at time of DCRS Clinic assessment (0-5; 0 is best function) | | | | | |
| average WHO Performance status  (Physician graded) | | 0.62 (±0.53) | 0.76 (±0.53) | 0.14 | 0.15 |
| Functional Activity Assessment Score at time of DCRS Clinic assessment (1-5; 1 is best function) | | | | | |
| average Functional Activity Assessment  (self-rated) | | 2.42 (±1.18) | 2.54 (±1.32) | 0.12 | 0.75 |

†symptoms scores: each patient was given a value of 1 for the presence of a symptom and 0 if they did not describe it. For ‘any breathlessness’ and ‘any cognitive symptom’ values are out of a total of 3 or 4 respectively (patients may describe breathlessness under up to three conditions and up to four possible cognitive symptoms). All values represent the ‘mean’ value for symptom score within the ‘hospitalised’ or the ‘community’ group.
