# Supplementary material for: Comprehensive clinical assessment identifies specific neurocognitive deficits in working-age patients with long-COVID
Source: PLoS One. 2022 Jun 10;17(6):e0267392. doi: 10.1371/journal.pone.0267392 (PMC9187094; doi:10.1371/journal.pone.0267392)
Supplement: S2 Table — (DOCX) [file pone.0267392.s003.docx]

**Supplementary Table 2 – WHO Performance Scale Acutely and at DCRS Clinic Assessment**

| WHO Performance Status Acutely | Number (%) /  Median (IQR) | WHO Performance Status at DCRS | Number (%) /  Median (IQR) | Significance of change in status |
| --- | --- | --- | --- | --- |
| 0 | 0 | 0 | 67 (33%) | P < 0.0001 |
| 1 | 25 (12.2%) | 1 | 131 (64%) |  |
| 2 | 92 (44.9%) | 2 | 7 (3%) |  |
| 3 | 78 (38%) | 3 | 0 |  |
| 4 | 10 (4.9%) | 4 | 0 |  |
| 5 | 0 | 5 | 0 |  |
| Median (IQR) | 2 (2-3) |  | 1 (0-1) | Decrease by >1 |

| WHO Performance Status | Description |
| --- | --- |
| 0 | Fully active, able to carry on all pre-disease performance without restriction |
| 1 | Restricted in physically strenuous activity but ambulatory and able to carry out work of a light to sedentary nature, e.g. light house work, office work |
| 2 | Ambulatory and capable of all self-care but unable to carry out any work activities. Up and about more than 50% waking hours |
| 3 | Capable of only limited self-care, confined to bed or chair more than 50% of waking hours |
| 4 | Completely disabled. Cannot carry on any self-care. Totally confined to bed or chair |
| 5 | Dead |
