# Supplementary material for: Comprehensive clinical assessment identifies specific neurocognitive deficits in working-age patients with long-COVID
Source: PLoS One. 2022 Jun 10;17(6):e0267392. doi: 10.1371/journal.pone.0267392 (PMC9187094; doi:10.1371/journal.pone.0267392)
Supplement: S3 Table — (DOCX) [file pone.0267392.s004.docx]

**Supplementary Table 3 – self rated Functional Activity Assessment (FAA) at DCRS**

| Functional Activity Assessment | Description | Number (%) |
| --- | --- | --- |
| 1 | Fully Fit | 43 (21%) |
| 2 | Fit for trade and restricted General or Military duties | 84 (41%) |
| 3 | Unfit for trade but fit for restricted General or Military Duties | 32 (16%) |
| 4 | Unfit for all but sedentary duties | 21 (10%) |
| 5 | Off all duties | 25 (12%) |
