# Supplementary material for: Comprehensive clinical assessment identifies specific neurocognitive deficits in working-age patients with long-COVID
Source: PLoS One. 2022 Jun 10;17(6):e0267392. doi: 10.1371/journal.pone.0267392 (PMC9187094; doi:10.1371/journal.pone.0267392)
Supplement: S4 Table — (DOCX) [file pone.0267392.s005.docx]

**Supplementary Table 4 – Additional clinical investigation results**

| Parameter | Normal range | Value (IQR) | Abnormal/tested  (%) | Abnormal /total (%) |
| --- | --- | --- | --- | --- |
| Bloods (all) | | | | |
| Hb | F 115-160; M130-175 g/L | 152 (146-158) | 1 (0.5%) = 179 g/L | 0.5% |
| WCC | 4-11 X 10^9^/L | 5.56 (4.85-6.66) | 15 (7%), 14x 2.5-4.0; 1 = 11.9 | 7% |
| Neutrophils | 2.0-7.5 X 10^9^/L | 3.0 (2.5-3.8) | 24 (12%), 23x 1.0-2.0; 1=8.9 | 12% |
| Lymphocytes | 1-4 X 10^9^/L | 1.9 (1.6-2.2) | 3 (1.5%) = 0.9 x 10^9^ | 1.5% |
| Platelets | 150-450 X 10^9^/L | 244 (217-284) | 0 |  |
| CRP | 0-10 mg/L | 1.2 (0.7-2.1) | 1 (0.5%)† | 0.5% |
| ESR | 1-20 mm/hr | 2 (2-5) | 2 (1%) | 1% |
| Urea | <7 mmol/L | 5.2 (4.5-6.1) | 18 | 9% |
| Creatinine | F 50-98; M 64-111 umol/L | 82 (73-92) | 2 (1%) | 1% |
| eGFR | >90 mL/min | NA | 44/205 (22%) | 22% |
| Alb | 35-52 g/L | 44 (42-47) | 1 (0.5%) =54 g/L | 0.5% |
| ALT | F <40; M<50 u/L | 31 (20-44) | 35 (17%) | 17% |
| AST | F <30; M<35 u/L | 33 (27-39) | 26 (13%) | 13% |
| Bilirubin | 0-21 umol/L | 13 (10-16) | 24 (12%) | 12% |
| Alk P | 40-150 u/L | 63 (53-75) | 1 (0.5%) | 0.5% |
| HbA1c | 20-41 u/L  (42-48 IGT; >48 T2DM) | 35 (33-38) | 5/205 (2%) 42-48 = IGT  4/205 (2%) >48 = T2DM | 2%, 2% |
| Observations (all) | | | | |
| HR |  | 84 (76-93) |  |  |
| BP |  | 120/80 (112/78-126/90) |  |  |
| SpO2 rest |  | 98% (98%-99%) |  |  |
| ABG (80 tests) | | | | |
| pH | 7.35 – 7.45 | 7.44 (7.42-7.46) | 22/80 (28%) >7.45 | 11% |
| HCO3 | 22-26 mmol/L | 25.2 (24.4-25.8) |  |  |
| lactate | 0-2.0 mmol/L | 0.8 (0.6-1.2) | 4/80 (5%) >2.0 | 2% |
| pO2 | >10.5 kPa | 13.6 (12.7-15.0) |  |  |
| pCO2 | 4.7 – 6.0 kPa | 4.9 (4.5-5.2) |  |  |
| CPET (all) | | | | |
| RER |  | 1.1 (1.1-1.2) |  |  |
| Borg RPE at peak |  | 17 (15-19) |  |  |
| Work (W) |  | 240 (213-270) |  |  |
| Work % pred | >85% of predicted | 96.9% (87.8%-110.5%) | 44 (21%) | 21% |
| VO2 l/min |  | 3.1 (2.6-3.4) |  |  |
| VO2 % pred | >85% of predicted | 109.3% (100%-124.9%) | 12 (6%) | 6% |
| VO2 at AT (% of peak predicted) | ≥40% of peak predicted | 40.0% (36.1%-42.9%) | 102 (50%) | 50% |
| VO_2_/heart rate (O_2_ pulse) |  | 100% (89.5%-112.5%) |  |  |
| VE/VCO2 at AT | <35.0 | 26.3 (24.4-28.2) | 6 (3%) | 3% |
| Breathing reserve at peak |  | 16.5% (6.9%-23.8%) |  |  |
| SpO_2_ at peak |  | 98.0% (96%-98%) |  |  |

†patient with acute COVID pneumonitis
