# Supplementary material for: Comprehensive clinical assessment identifies specific neurocognitive deficits in working-age patients with long-COVID
Source: PLoS One. 2022 Jun 10;17(6):e0267392. doi: 10.1371/journal.pone.0267392 (PMC9187094; doi:10.1371/journal.pone.0267392)
Supplement: S1 File — (DOCX) [file pone.0267392.s006.docx]

**Supplementary Methods**

*Baseline Observations*

Heart rate, blood pressure and peripheral oxyhaemoglobin saturation were acquired at admission to the ward using an IPM 8 Mindray Patient Monitor (Mindray UK Ltd, Huntingdon, UK).

*Height and Weight*

Weight was measured at admission to the ward using a SECA scale model 956-7021099 (Seca GmbH, Hamburg, Germany). Height was recorded from the patient’s primary healthcare record, from their most recent routine medical.

*Venous blood sampling*

Blood samples were drawn using 22-gauge Vacutainer® Eclipse devices (Becton Dickinson, Plymouth, UK) inserted into a vein in the participant’s antecubital fossa or forearm. Blood samples were saved directly into sealed, vacuum tubes (Vacutainer®, ®, Becton Dickinson, Plymouth, UK). Ethylenediaminetetraacetic acid (EDTA) tubes were used for plasma samples. Tubes with a clot activator and gel were used to collect serum samples. Blood samples were analysed in the pathology laboratory of the Nottingham University Hospitals NHS Trust, Queen’s Medical Centre. Results were accessed within one week, prior to the MDT which completed the assessment pathway.

*ECG*

ECGs were acquired following 2 minutes of supine rest using a GE Model MAC1600 ECG device (GE Healthcare, Chicago, US); 25 mm/s paper speed and 10mm/mV amplitude.

*Echocardiogram*

Echocardiograms were acquired with a Philips EPIQ 5 ultrasound scanner (Philips, Amsterdam, The Netherlands) by accredited echocardiographers working to the standard British Society of Echocardiography transthoracic echocardiogram dataset (2013).

*Cardiopulmonary exercise testing*

V̇O_2_ peak; peak workload; ventilatory efficiency (V̇E/V̇CO_2_); breathing reserve and oxygen pulse (V̇O_2_ peak/peak heart rate – which is correlated with cardiac stroke volume) were determined in a ramp exercise protocol to volitional fatigue on an upright cycle ergometer (Lode Corival, Lode BV, Groningen, The Netherlands). The test commenced with a two-minute resting recording, followed by two minutes of unloaded pedalling, then a progressive ramp protocol (starting at 25W with a ramp of 15-35 Watts/minute) to achieve a test lasting 8-12 minutes. Measurements of breath composition were performed by indirect calorimetry (Metalyzer® 3B Cortex Biophysik, Leipzig, Germany). Maximal testing was defined as a peak RER ≥1·10 and/or plateau in oxygen uptake despite increasing workload. Predicted peak V̇O_2_, predicted peak workload and predicted O_2_ pulse are based upon the Wasserman weight algorithm (31).

*Capillary blood gas assessment*

Capillary blood gas samples were taken from the earlobe at rest and immediately on cessation of exercise. Initial blood drops were discarded, and subsequent brisk-flowing drops collected in 125 μL heparinised-capillary tubes (safeCLINITUBES, Radiometer, Denmark) transferred to G4+ iStat cartridges and measured immediately with an iStat 1 Analyser (Abbott, Chicago, USA).

*Cross sectional imaging (HRCT, CTPA, CMR)*

High Resolution CT chest (HRCT) and/or Dual-Energy CT Pulmonary Angiography (DECTPA) scans were acquired using a dual-source CT (Siemens SOMATOM Drive, Siemens Healthineers, Erlangen, Germany). Interspaced HRCT protocol consisted of inspiratory 1mm sections with 10mm gap, followed by expiratory 1mm section with 30mm gap. DECTPA (1mm reconstructed slice thickness) and perfusion map were analysed using Siemens Syngo, CT CE Lung Analysis post-processing software. CMR scans were performed on Siemens MR scanners at 1.5-3 Tesla (Siemens Medical Solutions, Erlangen, Germany). Assessment of myocardial mass, volumes, and ejection fraction was performed with precordial ECG gating and acquired during expired breath-hold. Images were acquired to generate a left and right ventricular short axis stack (slice thickness 7mm, interslice gap 3mm) using a steady state free precession sequence (echo time 1.5ms, repetition time 3.0ms, temporal resolution 47.84ms, flip angle of 60⁰) as previously described (32).(32). Native T1 maps were acquired for 5 LV slices using Shortened Modified Look Locker T1 Mapping sequences (ShMOLLI, Siemens) and late gadolinium imaging was acquired by giving a bolus injection of 0.1mmol/kg of gadolinium-based contrast agent (Gadovist) followed by a T1-weighted phase-sensitive inversion recovery sequence, using techniques previously described (33, 34).(33, 34). Image analysis was performed by four experienced cardiologists with at least 10 years of CMR experience using CVI 42 analysis software (Circle Cardiovascular Imaging Inc., Calgary, AB, Canada).

*Data Collection*

Study data were collected and managed using REDCap electronic data capture tools hosted at the University of Birmingham. REDCap (Research Electronic Data Capture) is a secure, web-based application designed to support data capture for research studies, providing: 1) an intuitive interface for validated data entry; 2) audit trails for tracking data manipulation and export procedures; 3) automated export procedures for seamless data downloads to common statistical packages; and 4) procedures for importing data from external sources (35).
